# A pig model exploring the postnatal hair follicle cycle

**DOI:** 10.3389/fcell.2024.1361485

**Published:** 2024-09-26

**Authors:** Shujuan Li, Quan Zou, Yao Jiang, Yi Wang, Xiangdong Ding

**Affiliations:** State Key Laboratory of Animal Biotech Breeding, National Engineering Laboratory for Animal Breeding, Laboratory of Animal Genetics, Breeding and Reproduction, Breeding and Reproduction, Ministry of Agriculture and Rural Affairs, College of Animal Science and Technology, China Agricultural University, Beijing, China

**Keywords:** hair follicle cycle, anagen, catagen, telogen, pig model

## Abstract

**Introduction:**

The hair follicle (HF) is a micro-organ capable of regeneration. A HF cycle consists of an anagen, catagen and telogen. Abnormalities in the HF cycle can lead to many hair disorders such as hair loss. The pig is a good biomedical model, but there are few data on their HF cycle. The aim of this study was to classify the pig HF cycle and determine the feasibility of the pig as an animal model for human HF cycle.

**Methods:**

Skin samples from 10 different postnatal (P) days Yorkshire pigs was collected to determine the key time points of the first HF cycle in pig by H&E staining, immunofluorescence staining, q-PCR and western-blot.

**Results:**

By morphological observation and detection of markers at different stages, pig HF cycle was classified into three main periods - the first anagen until P45, catagen (P45–P85), telogen (P85–P100), and next anagen (>P100). In addition, we examined the expression of important genes *AE15*, *CD34*, *Versican*, *Ki67* et al. related to the HF cycle at different stages of pig HF, indicating that pig and human share similarities in morphology and marker gene expression patterns of HF cycle.

**Discussion:**

Our findings will facilitate the study of HF cycle and offer researchers a suitable model for human hair research.

## Introduction

The hair follicle (HF) is the most important skin appendage (Schneider et al., 2009), exhibiting extraordinary regenerative ability throughout life. The HF development process is complex and includes HF morphogenesis during the prenatal period and HF cycle development after birth (Williams et al., 2020; Choi, 2018). Most hair growth disorders in human are caused by HF cycle changes (Olsen et al., 2005). Therefore, research on the HF cycle has profound clinical significance. The structure of the mature HF mainly consists of the dermal papilla (DP), connective tissue sheath (CTS), outer root sheath (ORS), inner root sheath (IRS), and hair shaft (HS). The HF undergoes cyclic regeneration/growth (anagen), degeneration (catagen), and rest (telogen) (Muller-Rover et al., 2001; Castro and Logarinho, 2020; Paus and Foitzik, 2004; Sun et al., 2014). Catagen is the transitional phase follows anagen (Paus et al., 1994; Stenn and Paus, 1999), and for practical reasons, catagen has been divided into three stages: early catagen, mid-catagen, and late catagen. During the HF cycle, the morphological structure of the upper part remains unchanged, whereas that of the HF below the bulge shows cyclic morphological changes (Muller-Rover et al., 2001). These processes result from a series of inhibitory and activating factors influenced by the interactions between various cells in the skin microenvironment (Muller-Rover et al., 2001). Several classical genetic markers such as *CD34* (Poblet et al., 2006), *Versican* (Soma et al., 2005), *SOX9* (Vidal et al., 2005), *Wnt5a*, *Wnt10b* (Reddy et al., 2001), *SHH* (Song et al., 1999), *LHX2* (Kobielak et al., 2007), *LEF1* (Lee and Tumbar, 2012) et al. have been suggested to play an important role in the hair cycle, and studies on these genes can help to further demarcate the pig HF cycle and understand the molecular mechanisms.

Human hair research has shown many progresses in the morphological development and molecular mechanisms through the use of mouse models (Duverger and Morasso, 2014). However, fundamental differences exist between human and mouse HFs regarding their morphological structures, developmental mechanisms, and hair diseases (van Ravenzwaay and Leibold, 2004; Porter, 2003). Because of ethical and other limitations, the human skin is not readily available as an experimental material, limiting further research on human HF development. Therefore, a new, readily available animal model and structurally similar to human HFs is required to study HF development.

As a promising animal model, pigs (Sahoo et al., 2017) have a high degree of anatomical, genetic, and physiological similarity to humans, and experiments performed on pigs are more likely to predict an accurate picture of human pathophysiology than those performed on rodents (Meurens et al., 2012; Susin et al., 2017; Summerfield et al., 2015). The advantages of using pig models for exploring prenatal HF morphogenesis have been demonstrated (Jiang et al., 2021); however, data on HF cycle development in pigs are limited. Therefore, the study of pigs as model animals for HF development and HF diseases requires further exploration.

In this study, we have: 1) developed a method for categorizing and identifying stages of the postnatal HF cycle development in pigs. 2) classified the expression of key genes of the HF cycle in pigs, and 3) compared the structures and expression of marker genes of the HF cycle in pigs and humans.

## Materials and methods

### Ethical approval

All procedures involving laboratory animals were evaluated and authorized by the Institutional Animal Care and Use Committee (IACUC). The whole procedure for samples collected was carried out in strict accordance with the protocol approved by the IACUC (permit number DK996).

### Pig skin samples from postnatal stages

10 skin samples from Yorkshire pigs born in the same period at different postnatal stages (P10, P18, P20, P30, P40, P45, P62, P75, P85, and P100) were obtained from precisely localized dorsal skin areas, as described previously (Paus et al., 1999).

### Identification of HF cycle stages

Using a comprehensive guide for the recognition and classification of the distinct stages of the HF cycle, the HF cycle stages were identified based on the characteristics and gene expression of different periods. The morphologic criteria described should be used to determine the stages of a defined number of longitudinally cut HF per time point (e.g., 10 HF per time point) (Supplementary Figure S1). As described by Paus et al. (Paus et al., 1999) and Kloepper et al. (Kloepper et al., 2010), >2 TUNEL^+^ apoptotic cells in the hair bulb for early catagen; epithelial strand (ES), ball-shaped DP, hair club, germ capsule, thinner hair bulb and TUNEL^+^ apoptotic cells in ES for mid-catagen; thinner hair bulb matrix, brush-like hair club, shorter ES and apoptotic cells for late catagen; the second hair germ (HG) for telogen; onion-shaped DP, more hair bulb matrix, IRS, higher *Ki67* ^*+*^ cell for anagen. Hematoxylin and eosin (H&E) staining were used to show the morphological characteristics of each stage and immunofluorescence staining was used to detect the expression of marker genes at different stages to distinguish the different stages of the hair cycle.

### Localization of key genes at different periods of pig HF cycle

To further validate the periodization and to explore the expression of key genes for hair follicle cycle development at different periods of the pig HF cycle, we examined the expression of *CD34*, *AE15*, *Versican*, and *K14* genes associated with HF cycle development at different stages (P18, P45, P62, P75, P85, and P100) of the pig HF cycle. Gene expression and localization are presented by immunofluorescence staining. The information related to antibody details for immunofluorescent staining are in Supplementary Table S1.

### Key genes expression in different stages of pig HF cycle

Several genes such as *Wnt5a*, *Wnt10b* (Reddy et al., 2001), *SHH* (Song et al., 1999), *LHX2* (Kobielak et al., 2007), *LEF1* (Lee and Tumbar, 2012), *α-SMA* have been suggested to play an important role in the hair cycle. Therefore, we examined the expression of these genes in the anagen (P18), catagen (P62) and telogen (P85) to explore their effects on different periods of pig HF cycle development. Gene expression was detected by RT-PCR. In addition, we examined the expression of two classical marker proteins of the hair follicle, *SOX9* and *K14*, in different periods-the anagen (P18), catagen (P62), and telogen (P85) by Western blot. The antibody information details for Western blot are in Supplementary Table S1. The primer sequences are in Supplementary Table S2.

## Experimental techniques

### Hematoxylin-eosin staining

Skin tissue soaked in paraformaldehyde was cut into small pieces of approximately 1 × 3 mm along the direction of hair growth. The pieces were placed in an embedding box and soaked in formalin. Embedding boxes containing the tissues were rinsed in running water for 1 h, dehydrated in different gradients of ethanol (90%, 90%, 100%) for 1 h each time, then in 100% xylene (I→II→III) for 1 h each time. The paraffin wax was immersed in a thermostat at 55°C–65°C thrice for 1 h each time. The tissues were then embedded in paraffin. Paraffin blocks were cut into 2–3 µm slices using a slicer, spread in a 40°C–50°C water bath thermostat, fished out with a clean slide, and dried on a 37°C dryer to remove the moisture.

Paraffin sections were placed in a 60°C thermostat for 1 h and removed, washed thrice with xylene for 10 min each time, then washed thrice with anhydrous ethanol, stained with hematoxylin for 5 min, rinsed with running water for 10 min, stained with eosin for 5 min, washed briefly with alcohol, dehydrated with an ethanol gradient (70%, 90%, 100%) for 5 min each, washed thrice with xylene for 5 min each, and then covered with a coverslip using neutral resin.

### Immunofluorescence staining

Paraffin sections were removed from the oven at 60° for 1 h, washed three times with xylene for 10 min each time, washed with ethanol at 100%, 95%, and 80% concentrations for 10 min each, rinsed with running water for 5 min, washed once with phosphate-buffered saline (PBS; 15 min), and incubated with antigen restoration solution in the microwave oven for 5 min on high-power and then turned to low-power for 20 min. The sections were cooled for 1 h, washed thrice with PBS for 5 min/time, incubated with 5% sheep serum for 30 min, and incubated with primary antibody for 12 h at 4°C in a refrigerator. Paraffin sections were washed thrice with PBS, incubated with secondary antibody for 30 min, washed thrice with PBS, incubated with 4′,6-diamidino-2-phenylindole (DAPI, Solarbio, C0065) for 2 min, washed thrice with PBS, and then covered with an anti-fluorescence attenuation sealing solution. The samples were imaged using a TCS SP5 confocal microscope (Leica, Wetzlar, Germany). The numbers of cells and fluorescence intensity were analyzed using ImageJ (National Institute of Health, Bethesda, MD, United States) to quantify.

### Apoptosis detection

Paraffin sections were removed from the oven at 60°C for 1 h, washed thrice with xylene for 10 min each time, washed with anhydrous ethanol for 5 min, 90% ethanol for 2 min, 70% ethanol for 2 min, and deionized water for 2 min. Proteinase K without DNase was then added to the sample at 20ºC–37°C for 20 min. The TUNEL detection solution was added to the samples after washing with PBS thrice, and the samples were protected from light for 6 min. After washing thrice with PBS and adding DAPI, it was left for 2 min at room temperature. Finally, the sample was washed with PBS, and an anti-fluorescence attenuation sealing solution was added to the sample and observed under a confocal microscope.

### RNA extraction

Skin tissue was ground and put into a centrifuge tube containing TRIzol, to which 200 µL of chloroform was added, and centrifuged for 15 min (4°C, 12,000 r) after standing for 3 min at room temperature. The supernatant was then aspirated into a new tube, 500 µL of isopropanol was added, and the tube was left on ice for 20 min; after centrifugation (4°C, 12,000 r) the supernatant was poured off to retain the precipitate, which was washed twice using 1 mL of pre-cooled 75% alcohol, diluted with RNase-free double-distilled water (ddH_2_O), and finally the RNA was checked for quality before storage at −80°C.

### Quantification of mRNA with quantitative polymerase chain reaction (qPCR)

1) Total RNA Purification: Total RNA (1 μg) from the skin was added to 2 μL of 5×gDNA Eraser Buffer and 1 μL of gDNA Eraser (Takara Bio Inc., Tokyo, Japan) and then supplemented to 10 μL with RNase-free ddH_2_O. Purified RNA was obtained via PCR as per a specific procedure. 2) Reverse transcription of RNA: The following mixture was prepared: purification RNA (10 μL) in (1), Prime Script RT Enzyme Mix (1.0 μL), RT Prime Mix (1.0 μL), 5× PrimeScript Buffer 2 (4.0 μL), RNase Free dH_2_O (4.0 μL). The cDNA was obtained via PCR as per a specific procedure, and the cDNA was diluted 3-fold with RNase ddH_2_O. 3) q-PCR: The following mixture was prepared: 2 μL of cDNA, 1 μL of PCR Reverse Primer (10 μM), 1 μL of PCR Forward Primer (10 μM), and 6 μL of SYBR Green I Master. The mRNA expression was determined via q-PCR using the Roche Light Cycler 480 (Roche Diagnostics International AG, Rotkreuz, Switzerland).

### Western blotting

Skin tissues were added to protein lysate (RIPA) and protease inhibitor (PMSF; RIPA: PMSF = 100:1; Solarbio, Beijing, China), and the supernatant after centrifugation (4°C, 12,000×g, 5 min) was obtained for further analysis. The protein concentration was quantitatively analyzed using the Beyotime BCA Protein Concentration Assay Kit (Beyotime Biotechnology, Shanghai, China). A 10% sodium dodecyl-sulfate polyacrylamide gel electrophoresis (SDS-PAGE) gel was prepared as per the Beyotime kit, following which the proteins were separated into different bands via SDS-PAGE. After electrophoresis, the samples were transferred to a polyvinylidene difluoride membrane and blocked with a 10× mixture of tris-buffered saline and Polysorbate 20 (TBST) + milk powder to prepare a 5% milk powder blocking solution used for 2–3 h. Samples were washed five times with 1× TBST (5 min each time), incubated with the antibody (overnight at 4°C), washed five times with 1× TBST (5 min each time), and incubated with the secondary antibody (2 h at room temperature). The samples were washed five times with TBST and then photographed in a protein developer with a specific protein developer solution. The results were analyzed using ImageJ (National Institute of Health, Bethesda, MD, United States) to quantify protein expression.

## Results

### Anagen and catagen of postnatal pig HF

**FIGURE 1:**
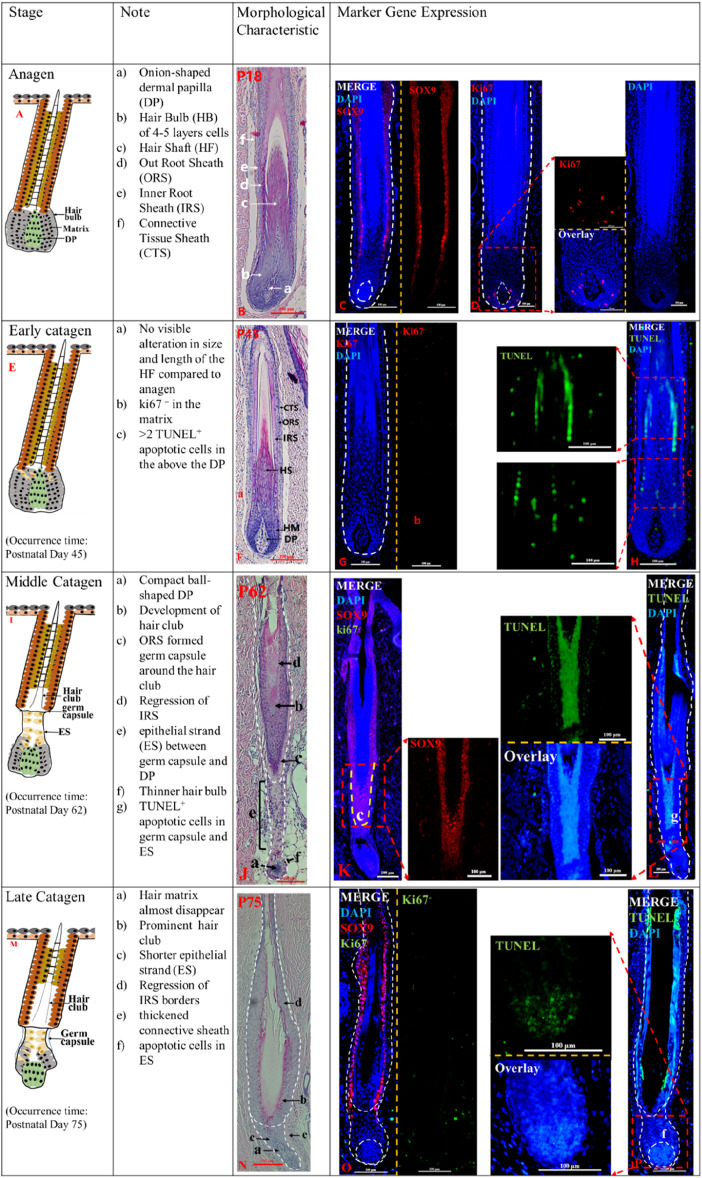
The anagen and catagen phases of postnatal pig HF. The identification of anagen (P18) **(A)**, early catagen (P45) **(E)**, mid-Catagen (P62) **(I)** and late Catagen (P75) **(M)**. The Note column summarizes simple basic parameters for the HF cycle obtained via hematoxylin and eosin staining and marker gene expression. The characteristics of the Anagen and Catagen in pig HF cycle were obtained via hematoxylin and eosin staining at P18 **(B)**, P45 **(F)**, P62 **(J)**, and P75 **(N)**. Gene expression in pig HF cycle at P18 **(C, D)**, P45 **(G, H)**, P62 **(K, L)**, and P75 **(O, P)** via immunofluorescence. Scale bars: 100 μm.

Anagen (<P45) and Early Catagen (P45-P62). Delineation of key time points in pig HF cycle based on morphological characteristics at different time points with marker gene detection (Figure 1; Supplementary Figure S1). Longitudinally cut HF at different days after birth revealed that the HF morphological structure has matured until 18 days after birth (P18), with the CTS, ORS, IRS, and hair shaft visible from the outer layer to the inner layer (Figure 1B). The *SOX9* ^*+*^ ORS is visible (Figure 1C), the hair bulb was filled with a hair matrix of four to five cell layers, and the DP expanded the hair bulb completely. *Ki67* ^*+*^ proliferating cells were detected in the hair matrix (Figure 1D; Supplementary Figure S2A). At P45, however, we found no *Ki67* ^*+*^ cells in the hair matrix (Figure 1G_b), instead TUNEL^+^ apoptotic cells were detected above the DP (Figure 1H_c and Supplementary Figure S2B), although the morphological structure of the HF does not seem to change significantly (Figure 1F). The results indicate that the matrix cells show signs of regression at P45.

Mid-catagen (P62-P75). The appearance of epithelial strand, hair club, and more TUNEL^+^ cells characterize the mid-catagen. At P62, the development of hair club, epithelial strand (ES) and the germ capsule around the hair club were the key features of the mid-catagen (Figure 1J). Germ capsule formation led to the constriction of the developing epithelial strand between the DP and germ capsule. The brush-like hair club (Figure 1J_b) was surrounded by *SOX9* ^*+*^ORS cell layers (Figure 1K). The decreasing hair matrix and proximal ORS cells formed the epithelial strand (Figure 1J_e) between the compact ball-shaped DP (Figure 1J_a) and germ capsule (Figure 1J_c). Degraded matrix leads to a thinner hair bulb (Figure 1J_f). Furthermore, the degraded IRS displayed sharply demarcated borders (Figure 1J_d). In addition, TUNEL^+^ cells were detected in the ES (Figure 1L_g; Supplementary Figure S2D), and no Ki67^+^ cells were observed in the matrix (Figure 1_K). These indicate that the HF is in the middle catagen.

Late catagen (P75-P85). The late catagen is characterized by the shorter ES, thinner matrix (almost disappear), and prominent hair club. At P75, the results of hematoxylin and eosin staining showed that HF morphology was altered compared to that at P62 (Figure 1N). The depressed ES (Figure 1N_c) was shorter and surrounded by a thickened connective sheath (Figure 1N_e). The thinner matrix was almost disappeared (Figure 1N_a). The *SOX9* ^*+*^ORS cells (Figure 1O) surrounded the brush-like hair club, and the IRS was unclear. Immunofluorescence staining showed TUNEL^+^ apoptotic cells (Figure 1P_f) in the ORS and DP.

### Telogen and next anagen of postnatal pig HF

**FIGURE 2:**
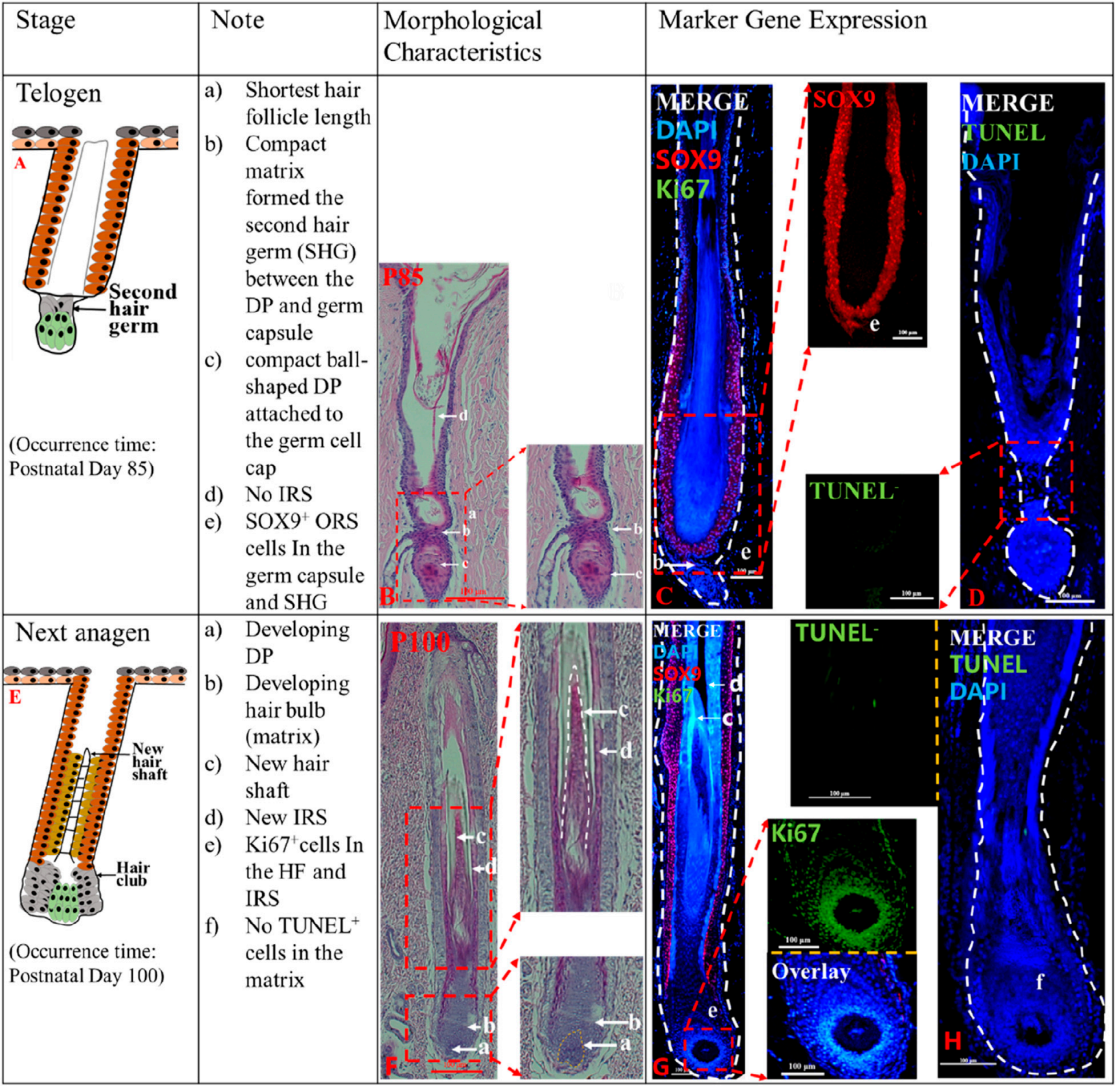
The telogen and the second anagen phases of postnatal pig HF. The identification of telogen (P85) **(A)** and second anagen (P100) **(E)**. The Note column summarizes simple basic parameters for the HF cycle obtained via hematoxylin and eosin staining and marker gene expression. The characteristic of the pig HF cycle was obtained via hematoxylin and eosin staining at P85 **(B)** and P100 **(F)**. Gene expression P85 **(C, D)**, P100 **(G, H)** in pig HF cycle at different stages via immunofluorescence. Scale bars: 100 μm.

Telogen (P85-P100). The telogen HF is easily recognizable because of the secondary hair germ (SHG). At P85, the lower HF length was the shortest (Figures 2A, B). The SHG was formed by compact matrix (Figure 2B_b), and a compact ball-shaped DP was closely attached to the SHG (Figure 2B_c). Telogen HF did not display any part of the IRS (Figure 2B_d). In addition, no TUNEL^+^ cells were detected in the SHG (Figure 2D), and a small number of *SOX9* ^*+*^ cells are present in the SHG (Figure 2C_e). The HF is in a state of relative silence in P85 with cellular inactivity.

Next Anagen (>P100). In this stage, the secondary hair germ proliferated and thickened to wrap around the developing DP (Figure 2F_a), and the HF matrix increased to form a newly expanded hair bulb (Figure 2F_b). New IRS (Figure 2F_d) and the hair shaft (Figure 2F_c) reappeared. Intense *Ki67* immunofluorescence in the hair matrix shows that the hair matrix is actively proliferating (Figure 2G_e; Supplementary Figure S2C), however, no apoptotic cells were detected (Figure 2H_f). The results showed that HF entered the new anagen phase at P100.

### Localization of key genes at different periods of HF cycle development in pig

**FIGURE 3:**
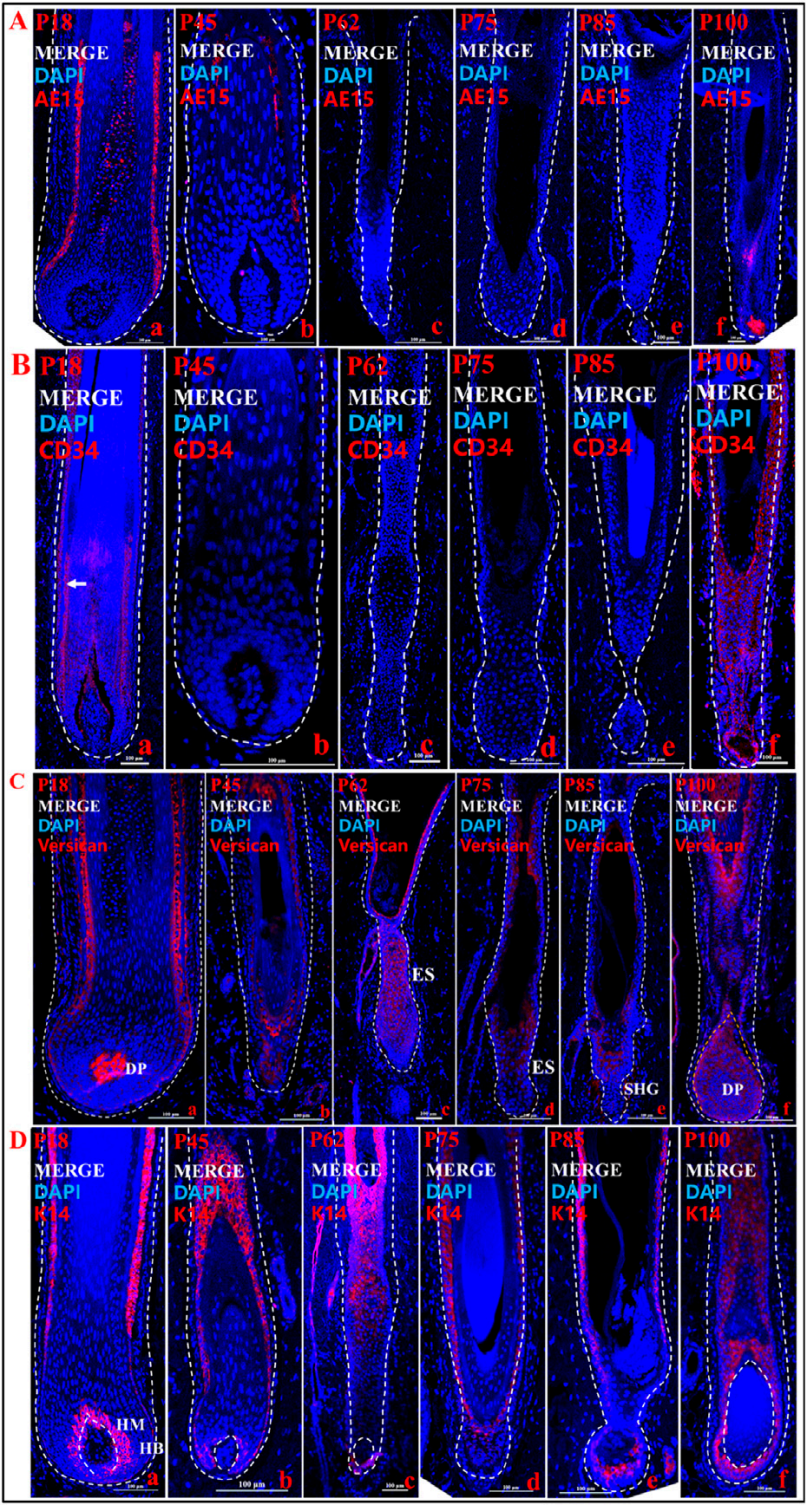
Localization of expression of key genes at different stages of hair follicle cycle development. **(A)** Immunofluorescence merge of AE15 in different time periods. **(B)** Immunofluorescence merge of CD34 in different time periods. **(C)** Immunofluorescence merge of Versican in different time periods. **(D)** Immunofluorescence merge of K14 in different time periods. a, b, c, d, e, and f show different periods. The blue fluorescence is DAPI, and the red fluorescence is the target gene. Scale bars: 100 μm.

In the above results, we initially classified the different stages of the pig HF cycle. To further determine the accuracy of the periodization and to explore the role of key genes in the cyclic development of pig HF, we examined the expression of *CD34*, *AE15*, and *Versican* at different stages (P18, P45, P62, P75, P85, and P100) of the pig HF cycle (Figure 3). We found that the marker gene for the IRS, *AE15*, was significantly expressed in P18 compared to P45 (Figure 3_a, b; Supplementary Figure S2E), but not in P62 (Figure 3A_c), P75 (Figure 3A_d) and P85 (Figure 3A_e), further suggesting a gradual regression of the IRS during HF degeneration. Interestingly, we found that *AE15* was expressed in the hair papilla and hair matrix during the new anagen phase (P100) (Figure 3A_f). *CD34* is expressed in the ORS in anagen (Figure 3B_a, f, arrowed), almost disappears in catagen (Figure 3B_b, c, d), and is not expressed during the telogen (Figure 3B_e). The *Versican* is expressed at different stages of the pig HF cycle, but there are differences in the level and location of expression. For example, *Versican* is specifically expressed in the DP in the anagen (Figure 3C_a, f), but in catagen and telogen, it is expressed mainly in the ES (Figure 3C_c, d) or SHG (Figure 3C_e) rather than in the DP. In addition, *K14*, as a classical marker of the hair follicle, was expressed in the ORS and the hair matrix at every stage (Figure 3D), and K14 immunofluorescence was intense in the hair matrix of anagen hair bulb (Figure 3_a, f).

From these findings, we clarified the spatial expression of key genes associated with HF cycle development on pig hair follicles cycle. At the same time, these results help division of the pig HF cycle.

### Key genes expression of the HF cycle in pig

**FIGURE 4:**
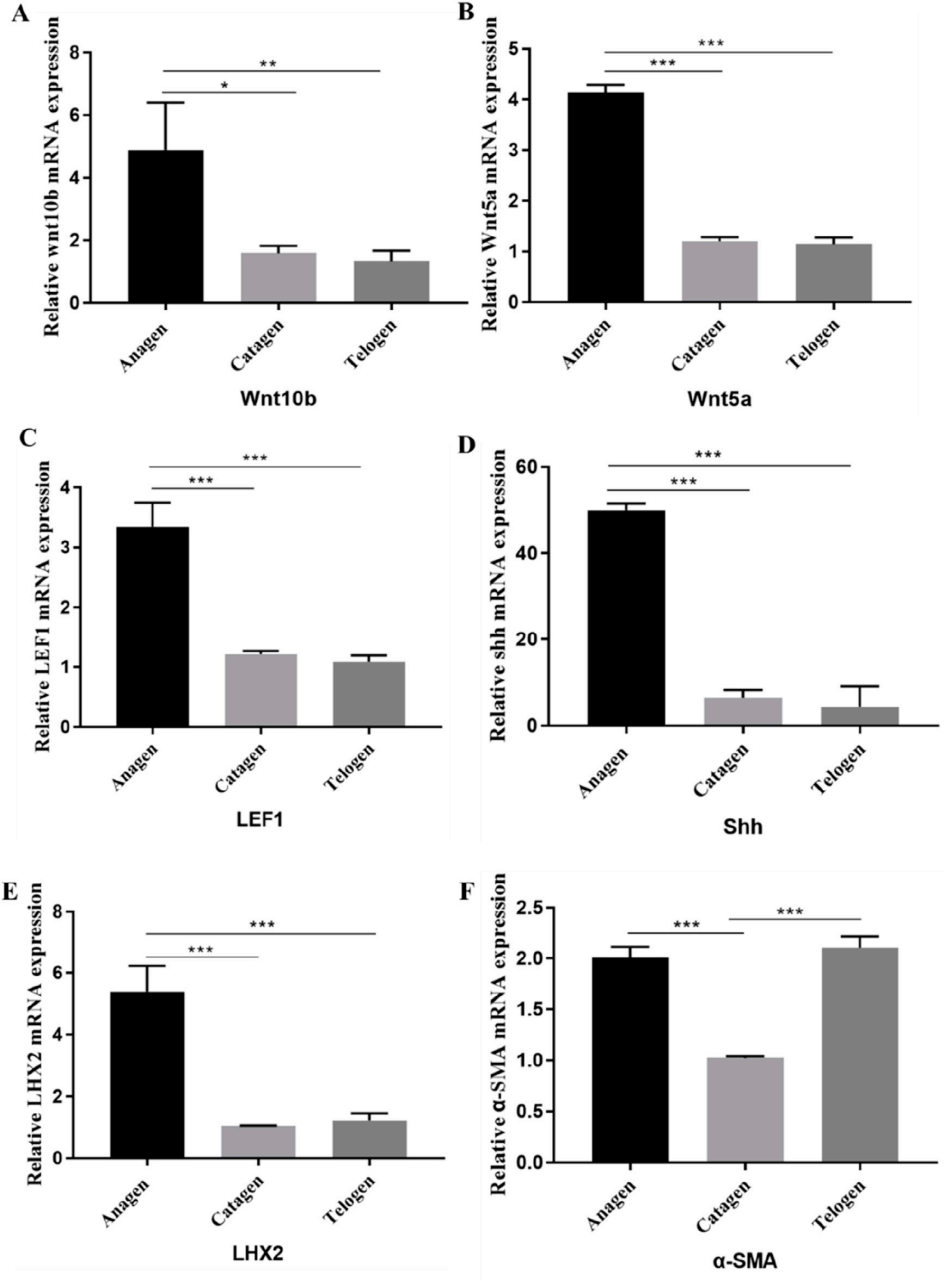
Expression of genes in the pig HF cycle. The mRNA expression of Wnt10b **(A)**, Wnt5a **(B)**, LEF1 **(C)**, SHH **(D)**, LHX2 **(E)**, α-SMA **(F)** in Anagen, Catagen and Telogen of HF cycle was detected by q-PCR. Data are presented as the mean ± SEM.*P< 0.05, **P< 0.01. ***P< 0.001 (Student’s t-test, n = 3 for each group).

To further investigate the expression of important genes affecting anagen, catagen and telogen in the pig HF cycle, some important genes related to HF cyclic development, such as *Wnt10b*, *Wnt5a*, *LEF1*, *SHH LHX2,* and *α-SMA* were selected for mRNA expression assays. The expression of *Wnt10b* (Figure 4A) and *Wnt5a* (Figure 4B) are significantly decreased when HF entered the catagen, and the expression of *LEF1* (Figure 4C), *SHH* (Figure 4D), and *LHX2* (Figure 4E) decreased significantly in the catagen compared to the anagen.

The results of gene expression during the telogen and anagen phases revealed that the expression of *Wnt10b*, *Wnt5a*, *LEF1*, *LHX2*, and *SHH* was significantly higher in anagen than in telogen (Figure 4). However, there was no significant difference in the expression of these genes between the catagen and telogen. It suggests that these genes may regulate the transition of hair follicles from anagen to catagen phase, rather than continuing to maintain the degenerative process. Additionally, the expression of *α-SMA* (Figure 4F) gene was significantly higher in anagen or telogen than in catagen, which may be related to the agglutination of DPs.

Furthermore, as classical markers of the hair follicle, *SOX9* and *K14* play important roles in HF cycle, and we also examined and quantified their protein expression using Western-bolt (Supplementary Figure S3). The results showed that the protein expression of *SOX9* and *K14* was significantly higher in the anagen than in the catagen and telogen. The result also helps to illustrate the changes of HF matrix in the HF cycle.

## Discussion

**FIGURE 5:**
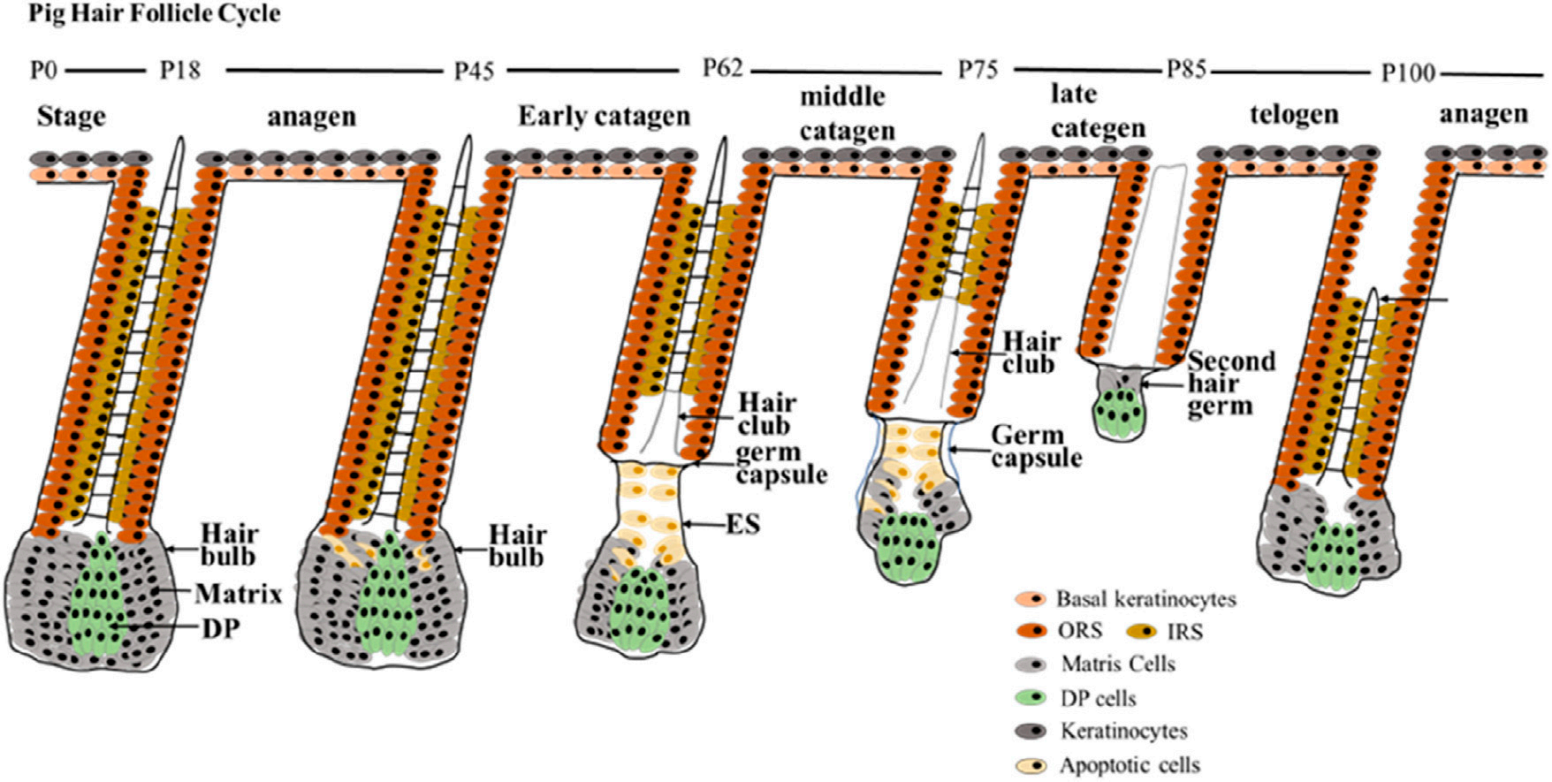
HF cycle development during postnatal in pigs. The first HF cycle of postnatal pigs has been summarized into three main periods. The anagen phase of postnatal pig HF is maintained up to P45; early catagen (P45–P62); mid-catagen (P62–P75); late catagen (P75–P85); telogen (P85–P100); and second anagen (> P100). Stages of the postnatal hair cycle are depicted. DP, dermal papilla; IRS, inner root sheath; ORS, outer root sheath.

The HF is an important appendage of the skin that shows extraordinary regenerative capacity during its life cycle, undergoing cyclic changes from growth (anagen) to degenerative phase (catagen) and rest (telogen) (Legue and Nicolas, 2005; Williams et al., 2020). According to our result, the HF cycle in pigs also goes through these three periods (Figure 5). After a period of growth, the HF enters the catagen phase, where the lower part of the HF undergoes apoptosis, and the DP degenerates and moves upwards. After undergoing a series of morphological changes, HF enters telogen phase and then continues the next cycle of growth. Based on the morphological characteristics and expression of marker genes in different HF regions at different time points, this study reported the first HF cycle development in pigs started at P45 and ended at P100. The time points for the first degradation, quiescence and regeneration of the pig HF cycle were identified. And the main characteristics of different stages of the pig hair cycle were described. Because pigs can reproduce continuously throughout the year, the follicular cycle stages of most newborn pigs do not coincide with those of mature pigs. Therefore, it is important to determine the follicular cycle pattern in pigs during their first year of life (Watson and Moore, 1990).

The human HF cycle can be divided into three periods, each period can be subdivided into different stages, anagen (anagen 1–6), catagen (early/mid/late-catagen), and telogen (Oh et al., 2016). Due to sample limitations, we did not observe all stages of HF cycle in pigs, however among the stages we observed, the similarities of morphology between pig and human HF were obtained. For instance, in morphology, the matrix and DP reduction (almond-shaped) are the signs of mid-catagen of human HF. And the epithelial strand, brush-like hair club and degraded or even disappeared IRS are important features in mid-catagen of human HF (Bodo et al., 2007; Oh et al., 2016; Commo and Bernard, 1997). These characteristics were also observed at mid-catagen (P62) of pig HF in this study. The characteristics of thinner matrix, prominent hair club, Shorter epithelial strand of late-catagen in human (Oh et al., 2016) were observed in late-catagen (P75) of pig HF in this study. The features of secondary hair germ, condensed small spherical-shape DP, and the almost disappeared matrix in telogen of human HF (Kloepper et al., 2010; KLIGMAN, 1959; Paus and Cotsarelis, 1999; Oh et al., 2016) were shown at telogen (P85) of pig HF. When the human HF is in the anagen phase, the CTS, ORS, IRS, and hair shaft is clearly visible (Oh et al., 2016), as we observed in anagen (P18 and P100) in pig.

Furthermore, the expression results of key genes or markers of pig HF cycle in this study showed similar patterns of marker expression of human HF cycle reported in the literature (Table 1). For instance, in anagen of human HF, *CD34* is expressed in ORS (Poblet et al., 2006), and our results indicate that *CD34* is expressed in the ORS of anagen in pig HF (Figure 3B). *Versican* immunofluorescence was intense in the DP of human anagen HF (Soma et al., 2005; Kishimoto et al., 1999). According to our results, the *Versican* is also highly expressed in the DP of anagen in pig HF (Figure 3C). High expression of IRS immunohistological markers can be used to differentiate anagen of human HF cycle (Oh et al., 2016). In our study, *AE15,* as a marker for the IRS of HF (Han et al., 2023), was significantly high expressed in anagen (P18) of pig HF (Figure. 3A; Supplementary Figure S2_e). In addition, *Ki67*, as a marker of anagen in human HF (Oh et al., 2016; Kloepper et al., 2010), was detected in anagen (P18) in pig HF.

**TABLE 1:** Expression of HF cycle markers in pig.

Periods	Region	Maker	Reference/human
Anagen	ORS	CD34^+^	(Poblet et al., 2006)
	IRS	AE15^+^	Commo and Bernard (1997)
	DP	Versican^+^	Soma et al. (2005)
	matrix	Ki67^+^	Oh et al. (2016)
Early catagen	epithelium above the DP	TUNEL^+^ Lower AE15	Commo and Bernard (1997), Botchkareva et al. (2006)
Mid-catagen	Epithelial strand	TUNEL^+^, AE15^−^	Lindner et al. (1997), Oh et al. (2016)
Late catagen	Shortened epithelial strand	TUNEL^+^, AE15^−^, CD34^−^	Oh et al. (2016), Poblet et al. (2006)
Telogen	Secondary hair germ (SHG)	TUNEL^−^ SOX9^+^	Oh et al. (2016), Kadaja et al. (2014)

Positive staining for apoptotic cells (by TUNEL immunofluorescence) in the regressing epithelium above the DP is used as a definitive immunohistological marker of early catagen in human (Botchkareva et al., 2006; Botchkareva et al., 2007). In our study, TUNEL^+^ cells were detected in the epithelium above the DP at early catagen (P45) in pig HF (Figure 1H_c; Supplementary Figure S2_b). Furthermore, downregulation of IRS immunohistological markers can be used to differentiate early catagen HFs from anagen HFs in human (Oh et al., 2016; Commo and Bernard, 1997), and in our study, the IRS marker *AE15* was significantly lower in the early catagen (P45) than anagen (P18) in pigs. For mid-catagen, IRS absence can be used to differentiate mid- to late catagen HFs from early catagen of human HF (Commo and Bernard, 1997). Consistently, our results show that IRS marker *AE15* can be detected in early catagen (P45) but is not expressed in mid-catagen (P62) in pig HF. TUNEL^+^ cells remained in the mid-catagen and late catagen in human (Oh et al., 2016; Lindner et al., 1997). Similarly, TUNEL^+^ cells were detected in the mid (P62) and late (P75) catagen of pig HF ((Figure 1L, P) in this study. In telogen of human HF, TUNEL^+^ cells are generally lacking in secondary hair germ (SHG) (Oh et al., 2016). Similarly, no TUNEL^+^ cell was detected in SHG of pig HF telogen in our study (Figure 2D). Besides, the change of *SOX9* expression at different periods can help the period delineation. *SOX9* ^*+*^ cells were detected at SHG in telogen of pig HF in this study. As reported, *SOX9* were located at the base of the follicle epithelium (Vidal et al., 2005) and SHG of human HF telogen (Kadaja et al., 2014). These results further illustrate that the human and pig hair follicle cycles share commonalities in gene expression patterns.

The HF cycle is regulated by many molecular signals. WNT signaling is essential for the HF cycle. *Wnt5a* and *Wnt10b* have been demonstrated to play important roles in HF morphogenesis and cyclic development, and *Wnt10b* is expressed in epidermal cells near hair papilla cells during telogen-to-anagen transition (Reddy et al., 2001). *Wnt5a* is expressed in the DP, ORS, and IRS in the anagen phase. Wnt5a is required for *SHH* expression during HF development, suggesting it to be a target of SHH signaling (Reddy et al., 2001). In addition, the WNT signaling pathway can also regulate the function of DP. DP cells induce stem cell activation during hair regeneration (Mifude and Kaseda, 2015).

The *SHH* signaling pathway plays an important role in HF epidermal cell proliferation and promotes deeper HF growth (Wang et al., 2000) and abnormal HF morphology after anagen phase treatment in the absence of *SHH* (Song et al., 1999). Follicular stem cell activation is an important factor in inducing follicular regeneration, and *LHX2* maintains follicular stem cell activity such that wild-type augmented stem cells retain labeled and utilized stem cells more rapidly than LHX2 deficient stem cells. *SOX9* and *LHX2* have been shown to inhibit WNT antagonist signaling, thereby ensuring that HFs enter a normal growth state (Gat et al., 1998).

To verify the affecting of genes related to HF cycle development on pig HF, we selected these genes for expression assays at different periods of pig HF. The mRNA expression levels of *Wnt10b*, *Wnt5a*, *LEF1*, *SHH*, and *LHX2* were significantly downregulated when HF entered the catagen phase. This also suggests that downregulation of the WNT and *LEF1* signaling may be an important regulatory signal promoting the catagen phase, whereas downregulation of the *Wnt5a* gene may be influenced by the *SHH* gene (Malumbres and Barbacid, 2003). Changes in *LHX2* expression may be related to its ability to maintain HF stem cell activity (Andl et al., 2002). The expression of *Wnt10b*, *Wnt5a*, *LEF1*, and *SHH* genes was significantly higher in anagen than in catagen and telogen, however, there was no significant difference of these gene expression between the catagen and telogen. Suggesting that these genes may regulate the transition of hair follicles from anagen to catagen, rather than continuing to maintain the degenerative process.

In summary, in this study, we explored the different stages of the first HF cycle in postnatal pig by HF morphological features and marker gene expression. Our findings indicated that morphology and gene expression patterns at different stages of the pig HF cycle were similar to those in humans. This study is not only an important complement to the HF cycle development in postnatal pigs, it provides a new animal model to the study of research on complex hair diseases in human.

## Data Availability

The original contributions presented in the study are included in the article/Supplementary Material, further inquiries can be directed to the corresponding author.

## References

[B1] Andl T. Reddy S. T. Gaddapara T. Millar S. E. (2002). WNT signals are required for the initiation of hair follicle development. Dev. Cell 2 (5), 643–653. 10.1016/s1534-5807(02)00167-3 12015971

[B2] Bodo E. Tobin D. J. Kamenisch Y. Biro T. Berneburg M. Funk W. (2007). Dissecting the impact of chemotherapy on the human hair follicle: a pragmatic *in vitro* assay for studying the pathogenesis and potential management of hair follicle dystrophy. Am. J. Pathol. 171 (4), 1153–1167. 10.2353/ajpath.2007.061164 17823286 PMC1988866

[B3] Botchkareva N. V. Ahluwalia G. Shander D. (2006). Apoptosis in the hair follicle. J. Invest Dermatol 126 (2), 258–264. 10.1038/sj.jid.5700007 16418734

[B4] Botchkareva N. V. Kahn M. Ahluwalia G. Shander D. (2007). Survivin in the human hair follicle. J. Invest Dermatol 127 (2), 479–482. 10.1038/sj.jid.5700537 16946715

[B5] Castro A. R. Logarinho E. (2020). Tissue engineering strategies for human hair follicle regeneration: how far from a hairy goal? Stem Cells Transl. Med. 9 (3), 342–350. 10.1002/sctm.19-0301 31876379 PMC7031632

[B6] Choi B. Y. (2018). Hair-growth potential of ginseng and its major metabolites: a review on its molecular mechanisms. Int. J. Mol. Sci. 19 (9), 2703. 10.3390/ijms19092703 30208587 PMC6163201

[B7] Commo S. Bernard B. A. (1997). Immunohistochemical analysis of tissue remodelling during the anagen-catagen transition of the human hair follicle. Br. J. Dermatol 137 (1), 31–38. 10.1046/j.1365-2133.1997.17641854.x 9274622

[B8] Duverger O. Morasso M. I. (2014). To grow or not to grow: hair morphogenesis and human genetic hair disorders. Semin. Cell Dev. Biol. 25-2622-33, 22–33. 10.1016/j.semcdb.2013.12.006 PMC3988237 24361867

[B9] Gat U. Dasgupta R. Degenstein L. Fuchs E. (1998). *De novo* hair follicle morphogenesis and hair tumors in mice expressing a truncated beta-catenin in skin. Cell 95 (5), 605–614. 10.1016/s0092-8674(00)81631-1 9845363

[B10] Han H. Qin H. Yang Y. Zhao L. Shen T. Pang Q. (2023). Effect of overexpression of KLF4 on the growth and development of hair follicles in mice. Dev. Genes Evol. 233 (2), 137–145. 10.1007/s00427-023-00708-8 37561178

[B11] Jiang Y. Zou Q. Liu B. Li S. Wang Y. Liu T. (2021). Atlas of prenatal hair follicle morphogenesis using the pig as a model System. Front. Cell Dev. Biol. 9721979, 721979. 10.3389/fcell.2021.721979 PMC8529045 34692680

[B12] Kadaja M. Keyes B. E. Lin M. Pasolli H. A. Genander M. Polak L. (2014). SOX9: a stem cell transcriptional regulator of secreted niche signaling factors. Genes Dev. 28 (4), 328–341. 10.1101/gad.233247.113 24532713 PMC3937512

[B13] Kishimoto J. Ehama R. Wu L. Jiang S. Jiang N. Burgeson R. E. (1999). Selective activation of the versican promoter by epithelial-mesenchymal interactions during hair follicle development. Proc. Natl. Acad. Sci. U. S. A. 96 (13), 7336–7341. 10.1073/pnas.96.13.7336 10377415 PMC22086

[B14] Kligman A. M. (1959). The human hair cycle. J. Invest Dermatol, 33307–33316. 10.1038/jid.1959.156 14409844

[B15] Kloepper J. E. Sugawara K. AL-Nuaimi Y. Gaspar E. van Beek N. Paus R. (2010). Methods in hair research: how to objectively distinguish between anagen and catagen in human hair follicle organ culture. Exp. Dermatol 19 (3), 305–312. 10.1111/j.1600-0625.2009.00939.x 19725870

[B16] Kobielak K. Stokes N. de la Cruz J. Polak L. Fuchs E. (2007). Loss of a quiescent niche but not follicle stem cells in the absence of bone morphogenetic protein signaling. Proc. Natl. Acad. Sci. U. S. A. 104 (24), 10063–10068. 10.1073/pnas.0703004104 17553962 PMC1888574

[B17] Lee J. Tumbar T. (2012). Hairy tale of signaling in hair follicle development and cycling. Semin. Cell Dev. Biol. 23 (8), 906–916. 10.1016/j.semcdb.2012.08.003 22939761 PMC3496046

[B18] Legue E. Nicolas J. F. (2005). Hair follicle renewal: organization of stem cells in the matrix and the role of stereotyped lineages and behaviors. Development 132 (18), 4143–4154. 10.1242/dev.01975 16107474

[B19] Lindner G. Botchkarev V. A. Botchkareva N. V. Ling G. van der Veen C. Paus R. (1997). Analysis of apoptosis during hair follicle regression (catagen). Am. J. Pathol. 151 (6), 1601–1617. 9403711 PMC1858357

[B20] Malumbres M. Barbacid M. (2003). RAS oncogenes: the first 30 years. Nat. Rev. Cancer 3 (6), 459–465. 10.1038/nrc1097 12778136

[B21] Meurens F. Summerfield A. Nauwynck H. Saif L. Gerdts V. (2012). The pig: a model for human infectious diseases. Trends Microbiol. 20 (1), 50–57. 10.1016/j.tim.2011.11.002 22153753 PMC7173122

[B22] Mifude C. Kaseda K. (2015). PDGF-AA-induced filamentous mitochondria benefit dermal papilla cells in cellular migration. Int. J. Cosmet. Sci. 37 (3), 266–271. 10.1111/ics.12190 25482359

[B23] Muller-Rover S. Handjiski B. van der Veen C. Eichmuller S. Foitzik K. Mckay I. A. (2001). A comprehensive guide for the accurate classification of murine hair follicles in distinct hair cycle stages. J. Invest Dermatol 117 (1), 3–15. 10.1046/j.0022-202x.2001.01377.x 11442744

[B24] Oh J. W. Kloepper J. Langan E. A. Kim Y. Yeo J. Kim M. J. (2016). A guide to studying human hair follicle cycling *in vivo* . J. Invest Dermatol 136 (1), 34–44. 10.1038/JID.2015.354 26763421 PMC4785090

[B25] Olsen E. A. Messenger A. G. Shapiro J. Bergfeld W. F. Hordinsky M. K. Roberts J. L. (2005). Evaluation and treatment of male and female pattern hair loss. J. Am. Acad. Dermatol 52 (2), 301–311. 10.1016/j.jaad.2004.04.008 15692478

[B26] Paus R. Cotsarelis G. (1999). The biology of hair follicles. N. Engl. J. Med. 341 (7), 491–497. 10.1056/NEJM199908123410706 10441606

[B27] Paus R. Foitzik K. (2004). In search of the “hair cycle clock”: a guided tour. Differentiation 72 (9-10), 489–511. 10.1111/j.1432-0436.2004.07209004.x 15617561

[B28] Paus R. Handjiski B. Czarnetzki B. M. Eichmuller S. (1994). Biology of the hair follicle. Hautarzt 45 (11), 808–825. quiz 824-5. 10.1007/s001050050180 7822211

[B29] Paus R. Muller-Rover S. Van Der Veen C. Maurer M. Eichmuller S. Ling G. (1999). A comprehensive guide for the recognition and classification of distinct stages of hair follicle morphogenesis. J. Invest Dermatol 113 (4), 523–532. 10.1046/j.1523-1747.1999.00740.x 10504436

[B30] Poblet E. Jimenez F. Godinez J. M. Pascual-Martin A. Izeta A. (2006). The immunohistochemical expression of CD34 in human hair follicles: a comparative study with the bulge marker CK15. Clin. Exp. Dermatol 31 (6), 807–812. 10.1111/j.1365-2230.2006.02255.x 16981909

[B31] Porter R. M. (2003). Mouse models for human hair loss disorders. J. Anat. 202 (1), 125–131. 10.1046/j.1469-7580.2003.00140.x 12587927 PMC1571051

[B32] Reddy S. Andl T. Bagasra A. Lu M. M. Epstein D. J. Morrisey E. E. (2001). Characterization of Wnt gene expression in developing and postnatal hair follicles and identification of Wnt5a as a target of Sonic hedgehog in hair follicle morphogenesis. Mech. Dev. 107 (1-2), 69–82. 10.1016/s0925-4773(01)00452-x 11520664

[B33] Sahoo S. Baker A. R. Haskins I. N. Krpata D. M. Rosen M. J. Derwin K. A. (2017). Development of a critical-sized ventral hernia model in the pig. J. Surg. Res. 210115-123, 115–123. 10.1016/j.jss.2016.10.026 28457317

[B34] Schneider M. R. Schmidt-Ullrich R. Paus R. (2009). The hair follicle as a dynamic miniorgan. Curr. Biol. 19 (3), R132–R142. 10.1016/j.cub.2008.12.005 19211055

[B35] Soma T. Tajima M. Kishimoto J. (2005). Hair cycle-specific expression of versican in human hair follicles. J. Dermatol Sci. 39 (3), 147–154. 10.1016/j.jdermsci.2005.03.010 15871917

[B36] Song J. Oh S. P. Schrewe H. Nomura M. Lei H. Okano M. (1999). The type II activin receptors are essential for egg cylinder growth, gastrulation, and rostral head development in mice. Dev. Biol. 213 (1), 157–169. 10.1006/dbio.1999.9370 10452853

[B37] Stenn K. S. Paus R. (1999). What controls hair follicle cycling? Exp. Dermatol 8 (4), 229–236. ; discussion 233-6. 10.1111/j.1600-0625.1999.tb00376.x 10439219

[B38] Summerfield A. Meurens F. Ricklin M. E. (2015). The immunology of the porcine skin and its value as a model for human skin. Mol. Immunol. 66 (1), 14–21. 10.1016/j.molimm.2014.10.023 25466611

[B39] Sun P. Watanabe K. Fallahi M. Lee B. Afetian M. E. Rheaume C. (2014). Pygo2 regulates β-catenin-induced activation of hair follicle stem/progenitor cells and skin hyperplasia. Proc. Natl. Acad. Sci. U. S. A. 111 (28), 10215–10220. 10.1073/pnas.1311395111 24982158 PMC4104891

[B40] Susin C. Fiorini T. Lee J. de Freitas R. M. Chiu H. C. Prasad H. S. (2017). Sinus augmentation using a mini-pig model: effect of ceramic and allogeneic bone biomaterials. J. Clin. Periodontol. 44 (10), 1059–1066. 10.1111/jcpe.12766 28644556

[B41] van Ravenzwaay B. Leibold E. (2004). The significance of *in vitro* rat skin absorption studies to human risk assessment. Toxicol Vitro 18 (2), 219–225. 10.1016/j.tiv.2003.08.002 14757113

[B42] Vidal V. P. Chaboissier M. C. Lutzkendorf S. Cotsarelis G. Mill P. Hui C. C. (2005). Sox9 is essential for outer root sheath differentiation and the formation of the hair stem cell compartment. Curr. Biol. 15 (15), 1340–1351. 10.1016/j.cub.2005.06.064 16085486

[B43] Wang L. C. Liu Z. Y. Gambardella L. Delacour A. Shapiro R. Yang J. (2000). Regular articles: conditional disruption of hedgehog signaling pathway defines its critical role in hair development and regeneration. J. Invest Dermatol 114 (5), 901–908. 10.1046/j.1523-1747.2000.00951.x 10771469

[B44] Watson S. A. Moore G. P. (1990). Postnatal development of the hair cycle in the domestic pig. J. Anat. 1701-9, 1–9. PMC1257057 2254156

[B45] Williams R. Pawlus A. D. Thornton M. J. (2020). Getting under the skin of hair aging: the impact of the hair follicle environment. Exp. Dermatol 29 (7), 588–597. 10.1111/exd.14109 32358903

