# Supplementary material for: A pig model exploring the postnatal hair follicle cycle
Source: Front Cell Dev Biol. 2024 Sep 26;12:1361485. doi: 10.3389/fcell.2024.1361485 (PMC11464431; doi:10.3389/fcell.2024.1361485)
Supplement: Supplementary file 1 [file Table1.pdf]

Table S1. Antibody Information for immunofluorescent staining (IF) and Western blotting (WB).

Antibody	Catalogue numbers	Supplier	Dilution
SOX9	Ab185966	abcam	IF: 1:300<br>WB: 1:3000
KRT14	sc-53253	Santa Cruz	IF: 1:100<br>WB: 1:500
Ki67	9449	Cell Signaling Technology	IF: 1:300
Ki67	TW0001S	Abmart	IF: 1:100
AE15	sc-80607	Santa Cruz	IF: 1:200
CD34	MK95982S	Abmart	IF: 1:100
Versican	T58111S	Abmart	IF: 1:100
β-Tubulin	M20005	Abmart	WB: 1:5000
Alexa Fluor 488-labeled Goat Anti-Mouse IgG(H+L)	AO428	Beyotime	IF: 1:500
Alexa Fluor 488-labeled Goat Anti-Rabbit IgG(H+L)	A0423	Beyotime	IF: 1:500
Alexa Fluor 647-labeled Goat Anti-Mouse IgG(H+L)	A0473	Beyotime	IF: 1:500
Alexa Fluor 647-labeled Goat Anti-Rabbit IgG(H+L)	A0468	Beyotime	IF: 1:500
HRP-conjugated Affinipure Goat Anti-Mouse IgG(H+L)	SA00001-1	proteintech	WB: 1:1000
HRP-conjugated Affinipure Goat Anti-Rabbit IgG(H+L)	SA00001-2	proteintech	WB: 1:1000
