# Supplementary material for: A pig model exploring the postnatal hair follicle cycle
Source: Front Cell Dev Biol. 2024 Sep 26;12:1361485. doi: 10.3389/fcell.2024.1361485 (PMC11464431; doi:10.3389/fcell.2024.1361485)
Supplement: Supplementary file 2 [file Table2.pdf]

Table S2. RT-PCR Primer Sequences

Primer	species	Sequences (5’-3’)	number of bases
GAPDH _F	Sus scrofa	ACCCAGAAGACTGTGGATGG	20
GAPDH_R	Sus scrofa	ACGCCTGCTTCACCACCTTC	20
WNT10b_F	Sus scrofa	ATGCGAATCCACAACAACAGG	21
WNT10b_R	Sus scrofa	GGGTCTCGCTCACAGAAGTCG	21
WNT5a_F	Sus scrofa	CTGGCAGGACTTTCTCAAGG	19
WNT5a_R	Sus scrofa	GAGGTGTTATCCACCGTGCT	19
LEF1_F	Sus scrofa	TCTCACTCCCCTCATCACTT	20
LEF1_R	Sus scrofa	TAATCTGTCCAACACCACCC	20
LHX2_F	Sus scrofa	TCTCGGACCGCTACTACCTGC	21
LHX2_R	Sus scrofa	ACCCGTGGTCAGCATCTTGTT	21
SHH_F	Sus scrofa	AGCCTACAAGCAGTTTATCCC	21
SHH_R	Sus scrofa	AGTGGTGGCCGTCCTCAT	18
a-SMA_F	Sus scrofa	AGAACACGGCATCATCACCA	20
a-SMA_R	Sus scrofa	CCAGAGGCGTAGAGGGACAG	20
