# Supplementary material for: A pig model exploring the postnatal hair follicle cycle
Source: Front Cell Dev Biol. 2024 Sep 26;12:1361485. doi: 10.3389/fcell.2024.1361485 (PMC11464431; doi:10.3389/fcell.2024.1361485)
Supplement: Supplementary file 3 [file Image2.pdf]

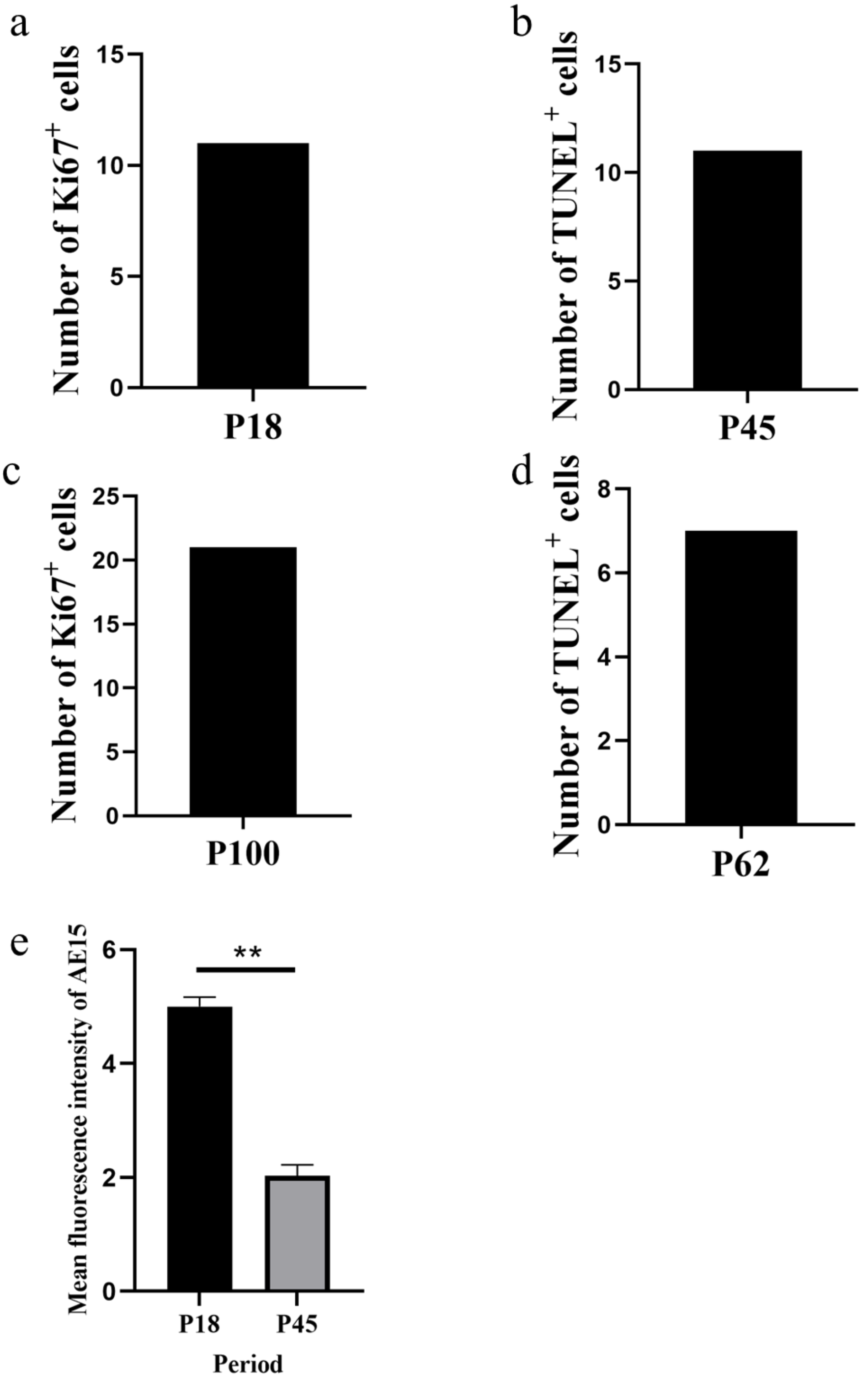

Figure S2. The quantitative information of immunofluorescence. (a) The quantitative of Ki67 in P18. (b) The quantitative of TUNEL in P45. (C) The quantitative of Ki67 in P100. (d) The quantitative of TUNEL in P62. (e) Quantification of the mean fluorescence intensity for P18 and P45. The results were analyzed and quantified using image J for gray value analysis. Data are presented as the mean ± SEM. **P<0.01. (student's t-test).
