# Supplementary material for: A pig model exploring the postnatal hair follicle cycle
Source: Front Cell Dev Biol. 2024 Sep 26;12:1361485. doi: 10.3389/fcell.2024.1361485 (PMC11464431; doi:10.3389/fcell.2024.1361485)
Supplement: Supplementary file 4 [file Image3.pdf]

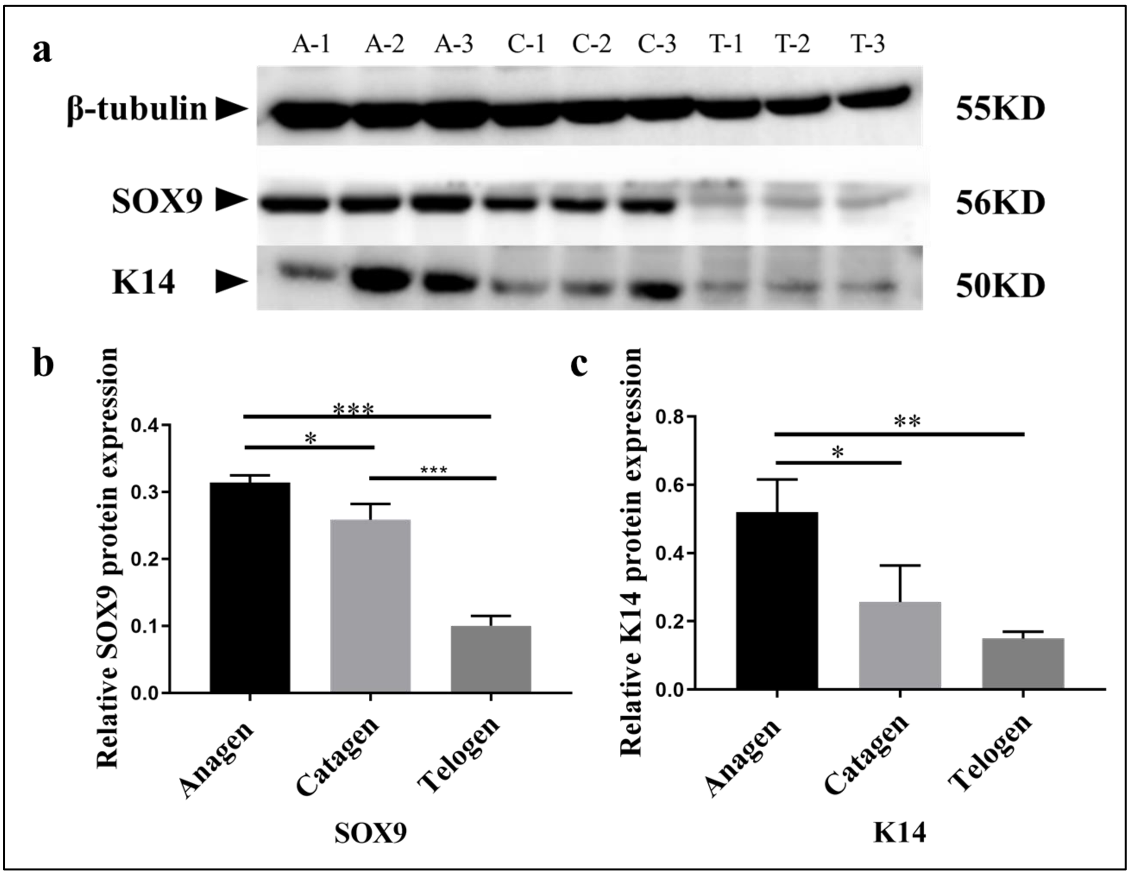

Figure S3. Expression of protein in the pig HF cycle. A: catagen, C: catagen, T: telogen. (a) Expression of SOX9 and K14 protein in different periods of pig HF cycle. (b) Quantification of SOX9 Protein. (c) Quantification of K14 Protein. The results were analyzed and quantified using image J for gray value analysis. Data are presented as the mean ± SEM.*P<0.05, **P<0.01. ***P<0.001 (student's t-test, n=3 for each group).
