# Supplementary material for: A pig model exploring the postnatal hair follicle cycle
Source: Front Cell Dev Biol. 2024 Sep 26;12:1361485. doi: 10.3389/fcell.2024.1361485 (PMC11464431; doi:10.3389/fcell.2024.1361485)
Supplement: Supplementary file 5 [file Image1.pdf]

The characteristics of different time in pig HF cycle were obtained via hematoxylin and eosin staining at P10, P18, P20, P30, P40, P45, P62, P75, P85 and P100. Scale bars: 100 mm.

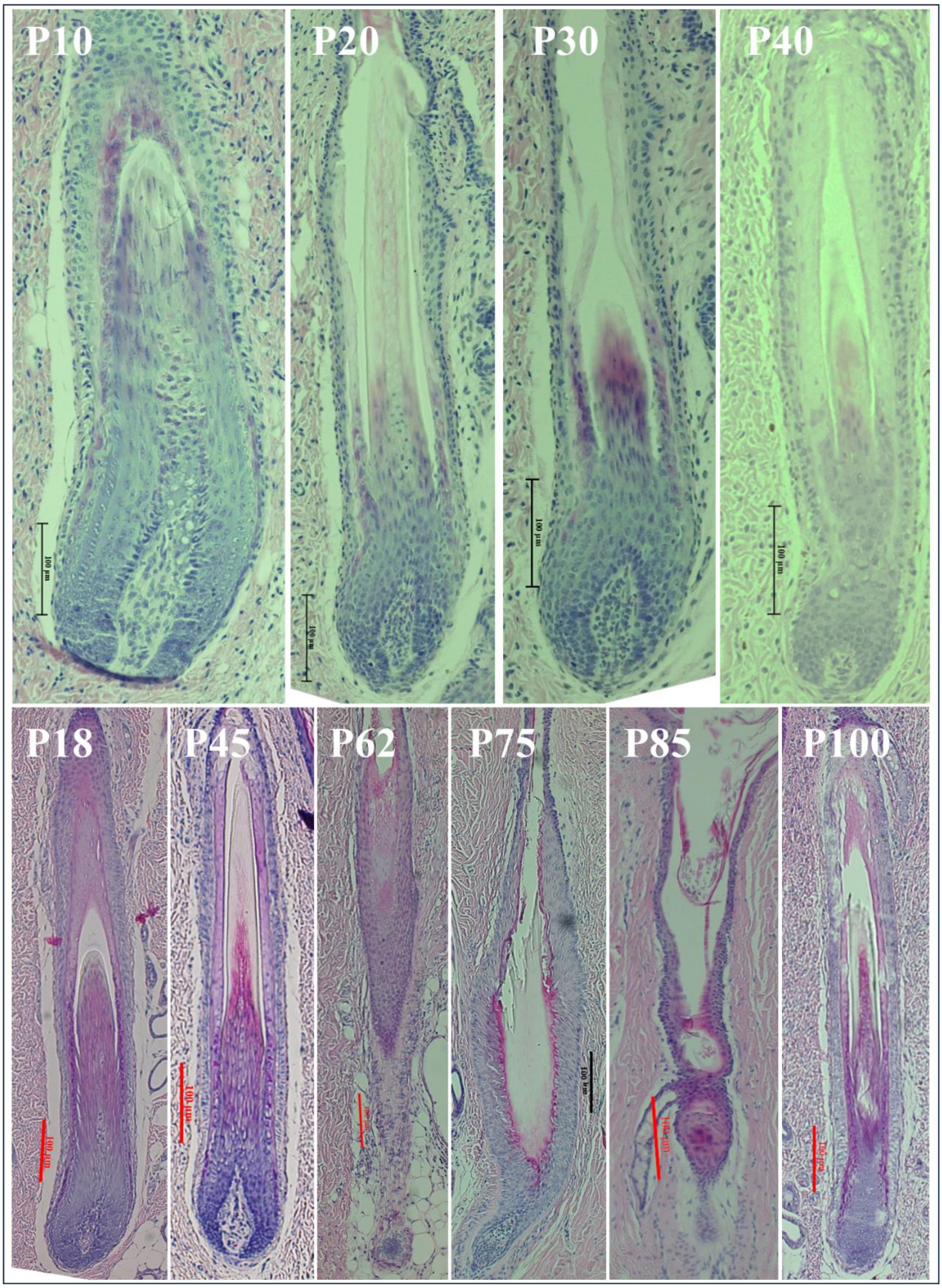

Figure S1. Hair follicle morphology at different time postnatal periods (P) in pigs. The characteristics of different time in pig HF cycle were obtained via hematoxylin and eosin staining at P10, P18, P20, P30, P40, P45, P62, P75, P85 and P100. Scale bars: 100 mm.
